# Bistable Multi‐Layer Triboelectric Nanogenerator for Harvesting Random and Ultra‐Low‐Frequency Vibration Energy with Increased Charge Transfer

**DOI:** 10.1002/advs.202505246

**Published:** 2025-06-24

**Authors:** Yi Guan, Xin Li, Zehan Wei, Mianxin Xiao, Zhihui Lai, Shuxiang Dong, Daniil Yurchenko, Shitong Fang

**Affiliations:** ^1^ Guangdong Key Laboratory of Electromagnetic Control and Intelligent Robots College of Mechatronics and Control Engineering Shenzhen University Shenzhen 518051 China; ^2^ National Key Laboratory of Green and Long‐Life Road Engineering in Extreme Environment (Shenzhen) Shenzhen University Shenzhen 518051 China; ^3^ Guangzhou Institute of Technology Xidian University Guangzhou 510555 China; ^4^ School of Materials Science and Engineering Peking University Beijing 100871 China; ^5^ Institute of Sound and Vibration Research University of Southampton Southampton S017 IBJ UK

**Keywords:** bistable, multi‐layer, ocean energy harvesting, self‐powered, triboelectric nanogenerator

## Abstract

Approximately 70% of the Earth’s surface is covered by seawater, making the ocean ideal for harvesting energy. Triboelectric nanogenerators (TENGs), due to their low cost and simple structure, are well‐suited for capturing ocean energy. However, their low charge transfer under weak inputs limits efficiency in harvesting random and ultra‐low‐frequency wave energy. This paper proposes a novel bistable multi‐layer TENG (BM‐TENG) to address this challenge for self‐powered wireless sensing and lighting. Simulations and experiments demonstrate that both in intra‐well and inter‐well motions, the bistable mechanism enhances the dynamic responses and thus the power output by up to 48%. Furthermore, the multi‐layer design within the constrained structure significantly boosts the power density. Experimental results show 730 V peak‐to‐peak open‐circuit voltage and 5 mW maximum power in a three‐layer BM‐TENG under the excitation of 0.6 Hz and 0.18 g. The normalized power density of the proposed device is 54.9 Wm^−3^·Hz^−1^, surpassing the state‐of‐the‐art results in literature. The application test shows that BM‐TENG can successfully power 296 LEDs for ocean warning lighting, and power Bluetooth wireless sensors for monitoring marine environmental variables. This work introduces a novel and highly efficient self‐powered sensing technique for advancements in marine Internet of Things (IoT) systems.

## Graphical abstract

To address the low energy conversion efficiency under random, ultra‐low‐frequency ocean excitations, this study introduces a novel bistable multilayer triboelectric nanogenerator (BM‐TENG) for efficient ocean energy harvesting and self‐powered marine sensing. The bistable mechanism enhances power output (up to 48%) via inter‐well and intra‐well oscillations, while the multilayer design increases power density beyond prior works (up to 54.9 Wm^−3^·Hz^−1^). It successfully powers 296 LEDs and Bluetooth sensors, validating its potential as a high‐efficiency solution for autonomous marine IoT applications.

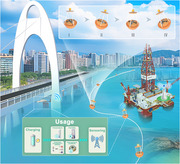

## 1 Introduction

With the rapid development of technology, the global demand for energy continues to rise,^[^ ^1^, ^2^ ^]^ and the environmental impact of combustion products from fossil fuels, as non‐renewable resources, has become a growing concern.^[^ ^3^, ^4^ ^]^ As a result, the utilization of clean energy has become a research focus. Ocean energy, as an emerging renewable resource, has attracted significant attention for its clean and pollution‐free characteristics.^[^ ^5^, ^6^, ^7^ ^]^ However, the ocean wave energy is influenced by factors such as seabed tectonic activity, lunar gravity, and wind forces,^[^ ^8^, ^9^, ^10^ ^]^ exhibiting ultra‐low frequencies (typically between 0.1–1 Hz) and randomness.^[^ ^11^ ^]^ These characteristics make it difficult for conventional energy harvesting structures to effectively capture such vibrational energy.

Existing energy harvesting technologies largely rely on linear electromagnetic generators (EMGs),^[^ ^12^, ^13^, ^14^ ^]^ piezoelectric generators (PEGs),^[^ ^15^, ^16^, ^17^, ^18^, ^19^ ^]^ triboelectric nanogenerators (TENGs)^[^ ^20^, ^21^, ^22^, ^23^, ^24^ ^]^ and hybrid generators.^[^ ^25^, ^26^, ^27^, ^28^, ^29^, ^30^ ^]^ However, EMGs and PEGs have low efficiency when harvesting ultra‐low‐frequency wave energy, and their complicated structural design, high manufacturing costs, regular maintenance requirements, limit their practical application. Therefore, the TENG, proposed by Wang and his team,^[^ ^31^, ^32^ ^]^ has emerged as a low‐cost, easy‐to‐maintain energy harvesting device that is well‐suited for low‐frequency vibrations, offering significant advantages in ocean energy harvesting. In addition to energy harvesting, TENG has shown great potential in health and environmental monitoring. TENG‐based sensors can monitor human health parameters (such as heart rate, respiration rate, and body temperature) in real‐time, offering advantages like portability, flexibility, and self‐powering, making them suitable for wearable devices.^[^ ^33^, ^34^ ^]^ Furthermore, TENG can also be used for environmental monitoring, such as detecting temperature, humidity, and gas concentrations, providing novel solutions for environmental protection and disaster early warning. Nevertheless, TENGs typically exhibit high output voltage but suffer from low output current, resulting in low overall output power that limits their effectiveness in practical application.^[^ ^35^ ^]^ Therefore, substantial linear structures have been proposed to increase the output power of the harvesters.^[^ ^12^, ^13^, ^36^ ^]^ These linear systems achieve maximum power output at their resonant frequency, which leads to significant limitations for ocean energy harvesting. The vibration frequency of the wave is variable, and the adaptability of the linear structure to ultra‐low‐frequency vibration is poor, so that such structures can not always work in the ideal frequency band. In response to the challenge of harvesting low‐frequency or random wave energy, extensive research has been conducted on nonlinear energy harvesters.^[^ ^37^, ^38^, ^39^, ^40^, ^41^, ^42^, ^43^, ^44^, ^45^ ^]^ By combining dynamic mechanisms with specialized structural designs, it has been found that nonlinear harvesting systems exhibit higher adaptability to random and low‐frequency excitations. Wang et al.^[^ ^46^ ^]^ designed a precise hybrid harvester based on dual quasi‐zero‐stiffness mechanism. The simulation and experimental results indicated that the performance of nonlinear system is better than that of linear system. Zhao and Ouyang^[^ ^47^ ^]^ proposed a nonlinear TENG composed of magnets and springs, which improves the output performance of low frequencies, meanwhile broadens the working frequency range compared with linear system. In addition, in previous ocean wave energy harvesting studies, scholars have proposed numerous novel and flexible structures, including the pendulum^[^ ^48^, ^49^, ^50^ ^]^ and spherical ones.^[^ ^51^, ^52^, ^53^ ^]^ In these studies, researchers have designed the housing of TENG in the shape of a marine buoy. These designs make the TENG highly sensitive to external vibrations, allowing it to operate continuously even under small amplitudes. However, the contact between charged spheres and plates typically occurs at a single point. This limited contact area results in insufficient frictional interaction and consequently low power generation efficiency. Such constrained performance makes these TENG unsuitable for efficiently harvesting wave energy. Ahmed et al.^[^ ^54^ ^]^ and Yang et al.^[^ ^55^ ^]^ designed TENGs with charged nylon spheres and copper spheres, respectively. They added multiple basic units for friction between the charged spheres and electrode surfaces. The increased friction area significantly boosts the output power through these multiple basic units.^[^ ^51^, ^52^, ^56^, ^57^, ^58^, ^59^ ^]^ Therefore, designing TENGs that can both improve its adaptiveness in harvesting random, ultra‐low‐frequency vibration energy, and boost structural charge transfer remains an unsolved challenge.

**Figure 1:**
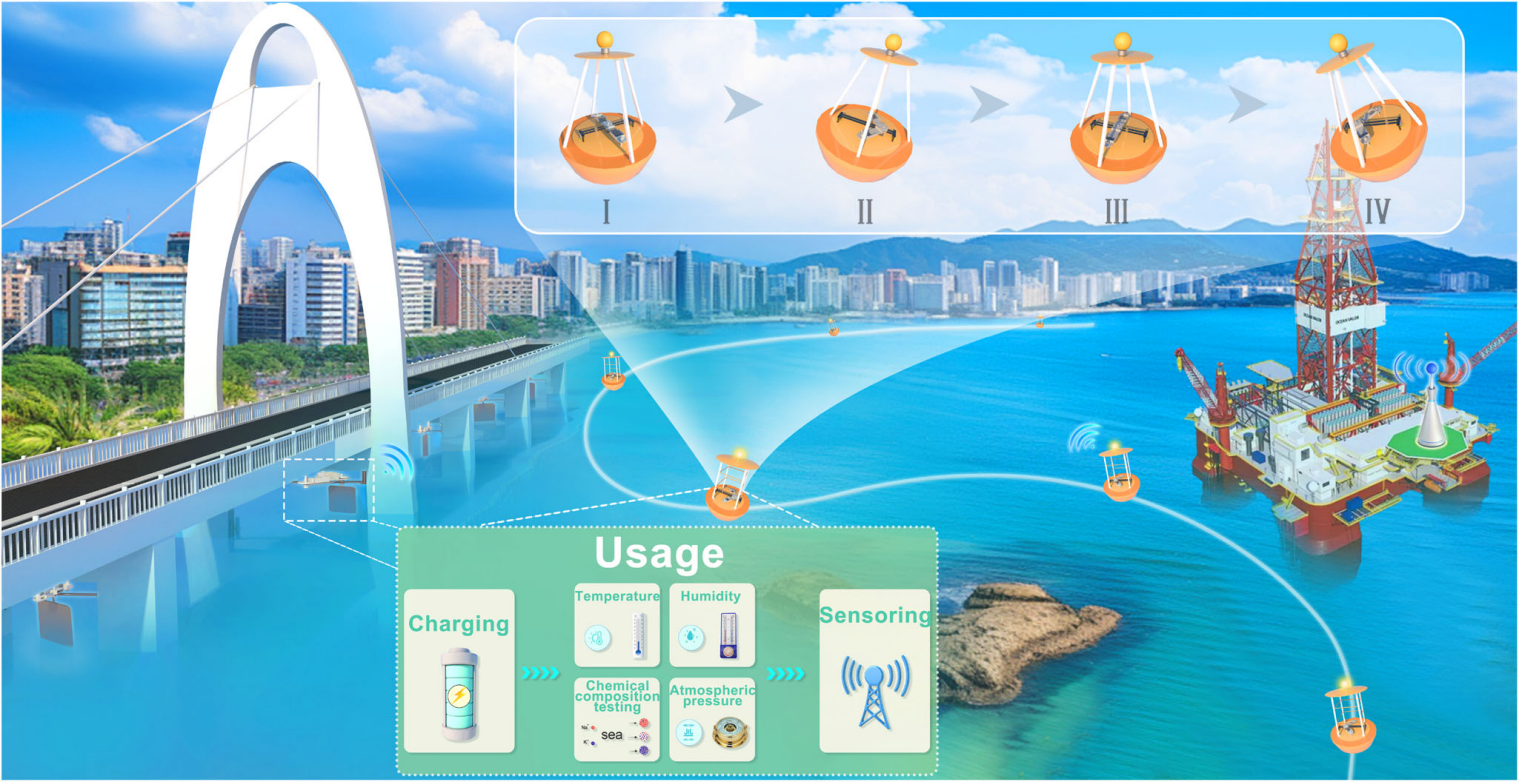
Schematic diagram of potential applications of BM‐TENG in the ocean. The schematic diagram image illustrates an ocean intelligent monitoring system based on BM‐TENG, covering urban bridges, ocean buoys, and offshore drilling platforms. BM‐TENG operates in two ways: a) wave‐driven internal structure vibration of the buoy (subfigures I‐IV) and b) wave flow‐induced vibration, harvesting energy to power environmental monitoring systems (temperature, humidity, chemical composition, atmospheric pressure). This establishes a self‐powered ocean sensing network, which can be widely applied to marine environmental monitoring systems and infrastructure safety management. a) The buoy continuously pitches under the impact of ocean waves. Under the influence of gravity, the mass block in the BM‐TENG tilts with the buoy, causing the mass block to drive the oscillating electrode to vibrate, thereby capturing the energy from the buoy's pitching motion. b) In the BM‐TENG, the force plate moves back and forth due to the impact of the waves, causing the oscillating electrode to vibrate and capture the energy from the wave's impact.

In this paper, an innovative bistable multi‐layer triboelectric nanogenerator is proposed for harvesting ultra‐low‐frequency wave energy. The design of BM‐TENG not only solves the issue of low efficiency in monostable or non‐stable systems but also increases the friction area compared with previous studies, thus improving the energy harvested from stochastic and ultra‐low‐frequency ocean environment, as shown in **Figure** **1**d. This paper discusses the dynamic responses of BM‐TENG under ultra‐low‐frequency periodic and stochastic excitation. A detailed parameter analysis of BM‐TENG is provided regarding different spring stiffness, equilibrium point positions, and excitation levels, thus offering parameter selection guidance under actual operating conditions. The experiment indicates that the BM‐TENG can achieve an RMS output power of 0.94 mW at 0.6 Hz, and the charge transfer of 2.16 µC per single cycle, indicating 48% increase in output power and 121% increase in RMS voltage compared to the monostable system, respectively. Application tests show that the proposed BM‐TENG can achieve real‐time self‐powered temperature monitoring, and light 296 LEDs. The proposed self‐powered TENG device demonstrates high application potential in ocean warning lighting devices and self‐powered micro‐sensors, which is of significance for the development of future ocean‐based IoT systems.

## 2 Results and Discussion

### 2.1 Design and Working Principle of the BM‐TENG

**Figure 2:**
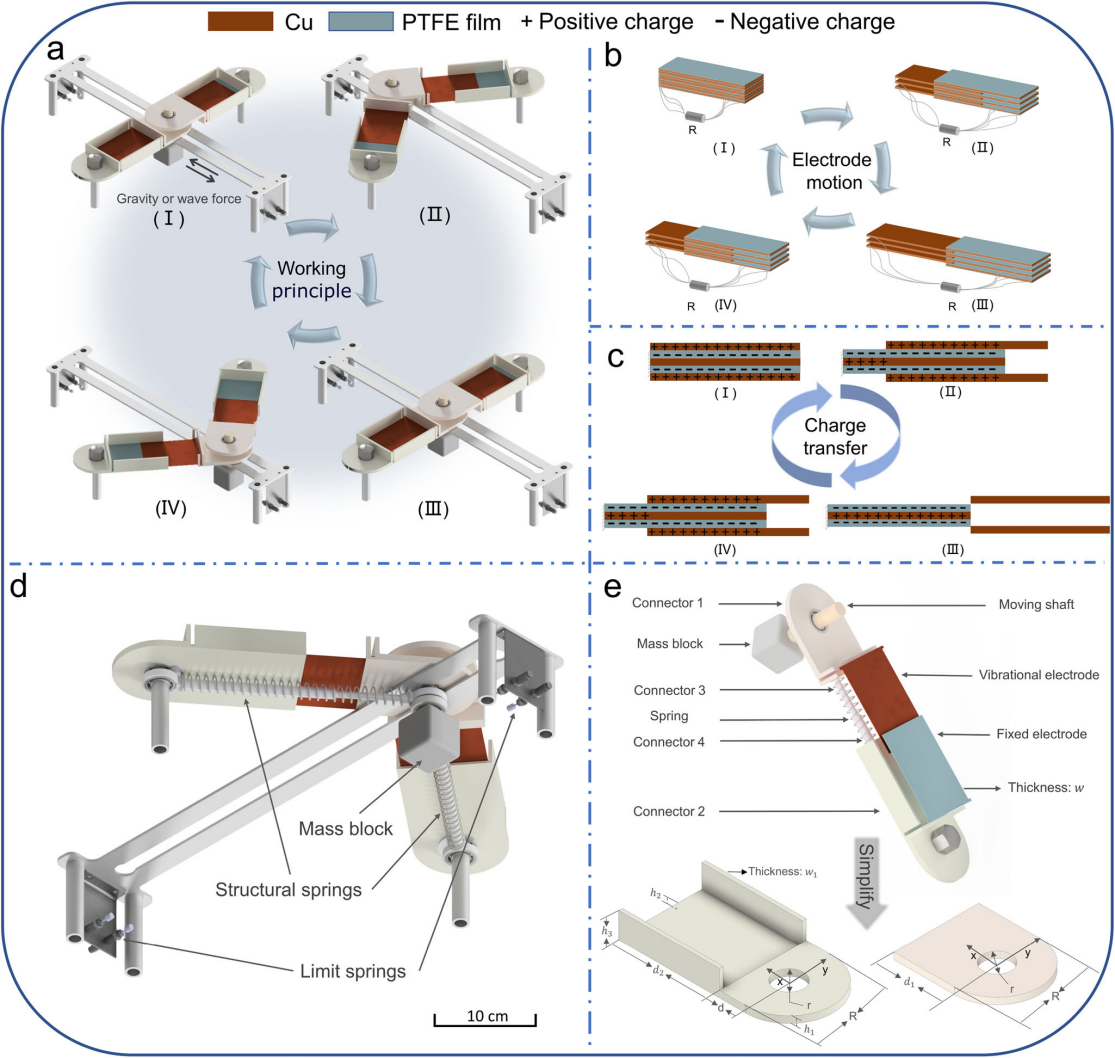
The configuration and working principle of BM‐TENG. a) The working process of BM‐TENG under gravity or wave force. b) Working principle of multi‐layer electrode plates. c) The process of charge transfer. d) The back and spring structure of the BM‐TENG. e) The specific structure of the electrode. (Spring assembly, connectors, electrode plates and mass block.).

As shown in **Figure** **2**, the BM‐TENG consists of three primary components: the support base, the energy harvesting structure and the spring structure. The support base consists of a stainless steel plate and two fixed shafts. The plate restricts the motion of the moving shaft to reciprocating linear movement along the groove. The connector 2 is connected with the fixed shaft through bearings in order to avoid deformation. Both ends of the plate are equipped with limiting devices comprising limit plates and hydraulic buffer springs. These devices restrict the maximum displacement of the moving shaft, ensuring that the fixed and oscillating electrode plates remain partially engaged and do not fully separate.

In Figure 2d,e, the energy harvesting structure includes the moving shaft (comprising bearings, a shaft, and a mass block), fixed electrodes, oscillating electrodes, connector 1, and connector 2. Connector 1 connects the moving shaft with the oscillating electrode plates. Similarly, connector 2 enables the rotational motion of the fixed electrode plates around the fixed shaft. Consequently, when the moving shaft moves under an external force, the fixed electrode plates rotate only about the fixed shaft, while the oscillating electrode plates rotate about the moving shaft and move toward the fixed shaft at the same time. Therefore, the motion of the moving shaft from the center to the end represents a process in which the electrode separation distance progressively increases, whereas the reverse motion indicates a gradual decrease of separation distance.

The spring structure comprises a spring assembly, connectors 3 and 4. Connectors 3 and 4 are connected to the moving and fixed shafts through bearings. The springs are mounted externally to connectors 3 and 4, with their ends securely attached to these connectors. Additionally, connectors 3 and 4 stabilize the springs under compressive loads, preventing buckling. In summary, spring assembly plays a crucial role in the system, and the properties of the spring itself completely determine the kinematic characteristics of the system under the bistable mechanism.

In Figure 2a–c, the entire motion process of the system can be clearly observed. Figure 2b,c depicts the movement process of the electrodes and the transfer of charges. When the moving shaft is positioned in the middle of the groove, the oscillating electrode and the fixed electrode completely overlap. At this moment, positive charges are concentrated on the surface of the oscillating electrode that rubs against the PTFE membrane. As the oscillating electrode and the fixed electrode separate, due to electrostatic induction, the positive charges in the oscillating electrode spontaneously transfer through the circuit to the copper plate surfaces to balancing the electric field. When the oscillating electrode and the fixed electrode are completely separated, all positive charges are concentrated on the copper plate surfaces. Since the negative charges in the PTFE film are concentrated on the surface that rubs against the oscillating electrode, the electric field near the friction surface is larger compared to other areas. Therefore, when the oscillating electrode and the fixed electrode come into contact again, the positive charges on the copper plates in the fixed electrode transfer back to the copper plate surfaces in the oscillating electrode. Therefore, when the system completes a cycle on one groove side, it presents the charges have transferred twice, forming alternating current. Additionally, if the moving shaft moves from one side of the groove to the other, the charges in the copper plates also transfer twice.

### 2.2 Theoretical Modeling

The Lagrangian dynamics equation is derived from Newton's second law,^[^ ^60^ ^]^ with its formula as follows:

(1) $$\frac{d}{dt}\left(\frac{\partial L}{\partial {\dot{q}}_{k}}\right)-\frac{\partial L}{\partial q_{k}}=\frac{\delta W_{i}}{\delta q_{k}}$$

(2) $$L=E_{k}-E_{p}$$

where *E*_*k*_ is the total kinetic energy of the system, *E*_*p*_ is the total potential energy of the system, *W*_*i*_ is the work done by non‐conservative forces, and *q*_*k*_ is the generalized coordinate. In BM‐TENG, the system's total kinetic energy *E*_*k*_ consists of the moving shaft *E*_*k*1_, oscillating electrode *E*_*k*2_, fixed electrode *E*_*k*3_, springs, and connectors. The potential energy consists of the elastic potential energy *E*_*p*1_ of the springs attached to the connectors 3 and 4, and the stopper spring. Notably, the spring in the limiting structure is not always compressed; it only gets compressed and stores elastic potential energy when the moving shaft reaches either end and contacts the spring.

The non‐conservative forces consist of two parts: external excitation *F*_*e*_ and friction force *F*_*f*_. Therefore, the work done by *F*_*e*_ and *F*_*f*_ are *W*_*e*_ and *W*_*f*_, respectively.

(3) $$W_{e}=F_{e}h,\;W_{f}=F_{f}x$$

(4) $$F_{e}=A\cos\left(\phi t\right),\;F_{f}=-\frac{\dot{x}}{|\dot{x}|}f_{f}$$

where *f*_*f*_ is the value of *F*_*f*_, which is a scalar, $-\frac{\dot{x}}{|\dot{x}|}$ represents the direction of *F*_*f*_, *A* is excitation amplitude, ϕ is excitation frequency. The electrostatic force between the PTFE films and aluminum can be neglected. Thus, the work done by each can be derived with respect to the generalized coordinates, then combined into the Lagrangian dynamics equation as:

(5) $$\frac{d}{dt}\left(\frac{\partial L}{\partial \dot{\theta }}\right)-\frac{\partial L}{\partial \theta }=\frac{A\cos\left(\phi t\right)L_{s}}{\cos^{2}\theta }-\frac{f_{f}L_{s}\dot{\theta }\left|\sin\theta \right|}{|\dot{\theta }|\cos^{2}\theta }$$

where *L* = *E*_*k*1_ + *E*_*k*2_ + *E*_*k*3_ − *E*_*p*1_ − *E*_*p*2_, and $\frac{\dot{\theta }}{|\dot{\theta }|}$ means taking only the sign of $\dot{\theta }$. This is due to the fact that the friction force direction is always opposite to the direction of the moving shaft's motion. *L*_*s*_ is the distance from the center of the moving shaft to the fixed shaft center when the moving shaft is in the middle position. ω is the derivative of the generalized coordinate rotation angle θ, i.e., the angular velocity.

This paper adopts the electrode‐dielectric in‐plane sliding model, where the open‐circuit voltage *V* and the transferred friction charge *Q* are closely related to the separation distance between two electrodes. The relationship can be described by the *V* − *Q* − *x* equation:

(6) $$V=-\frac{1}{C(x)}\times Q+V_{oc}\left(x\right)$$

where *V* is the output voltage. *C*(*x*) is the capacitance between the two electrodes. *Q* is the charge transferred between the two electrodes, and *V*_*oc*_(*x*) is the open‐circuit voltage. Although this equation is a predictive semi‐analytical model rather than an exact theoretical analysis, it allows the prediction of the approximate performance of the sliding friction model. According to Ohm's law $V=R\frac{dQ}{dt}$, integrating the Ohm's law into Equation (6) to derive the following equations:

(7) $$I=\sigma wv\frac{d}{Rw\varepsilon_{0}\varepsilon_{r}v-d}\left\{\frac{l}{l-vt}\exp\left[\frac{d}{Rw\varepsilon_{0}\varepsilon_{r}v}\ln\left(\frac{l-vt}{l}\right)-1\right]\right\}$$

Thus, the output voltage of the in‐plane sliding model can be expressed as:

(8) $$V=\sigma wvR\frac{d}{Rw\varepsilon_{0}\varepsilon_{r}v-d}\left\{\frac{l}{l-vt}\exp\left[\frac{d}{Rw\varepsilon_{0}\varepsilon_{r}v}\ln\left(\frac{l-vt}{l}\right)-1\right]\right\}$$

This equation represents a semi‐analytical model of the in‐plane sliding mode structure, which can be used to predict its electrical characteristics. The equation includes terms *v* and *vt*, indicating that the electrical properties of the system are influenced by the relative velocity and separation distance between the fixed electrode and oscillating electrode. The detailed derivation and formula are given in the Section S1 (Supporting Information).

In conclusion, coupling the dynamic and electrical model together, when there is external harmonic or random excitation, enables the prediction of energy conversion efficiency of the system. By adjusting the initial values of various parameters of the system, both the dynamic and electrical properties can be changed. The above equations are realized by MATLAB code, and the output power and output voltage under different parameters can be analyzed in detail through modeling. These models provide a theoretical basis for the dynamics research of the whole paper.

### 2.3 Dynamic Responses of the BM‐TENG under Different Excitation Amplitudes and Frequencies

**Figure 3:**
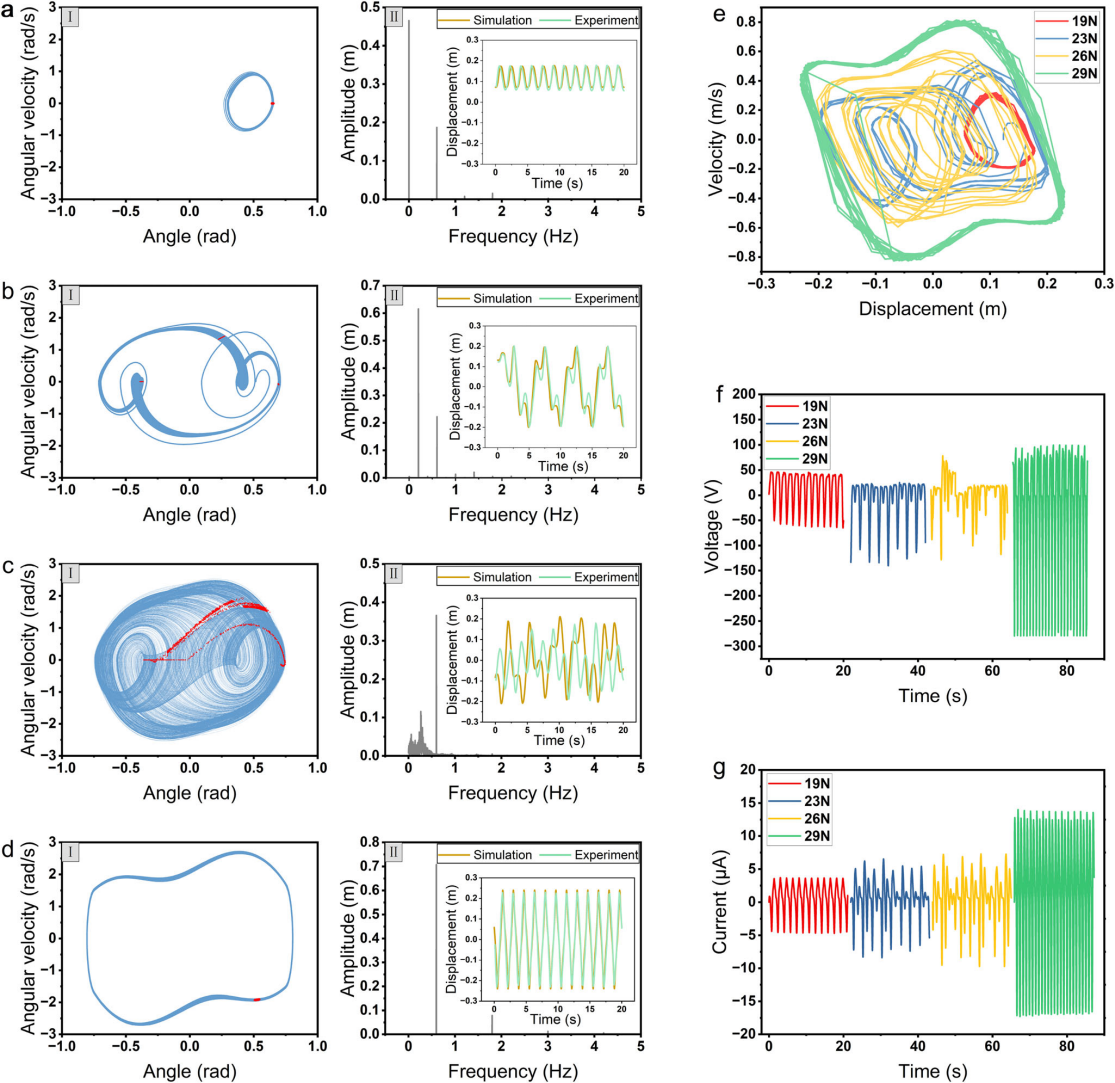
Simulation results of the Poincaré map of the motion on its phase portrait, Fast Fourier Transform, and displacement of moving shaft under excitation amplitudes of a) 19 N, b) 24 N, c) 25 N, and d) 29 N. e) Experimental results of the phase portrait of the BM‐TENG under 19 N, 23 N, 26 N and 29 N. Experimental results of the open‐circuit voltage (f) and short‐circuit current (g) of the BM‐TENG under different excitation amplitudes.

**Figure 4:**
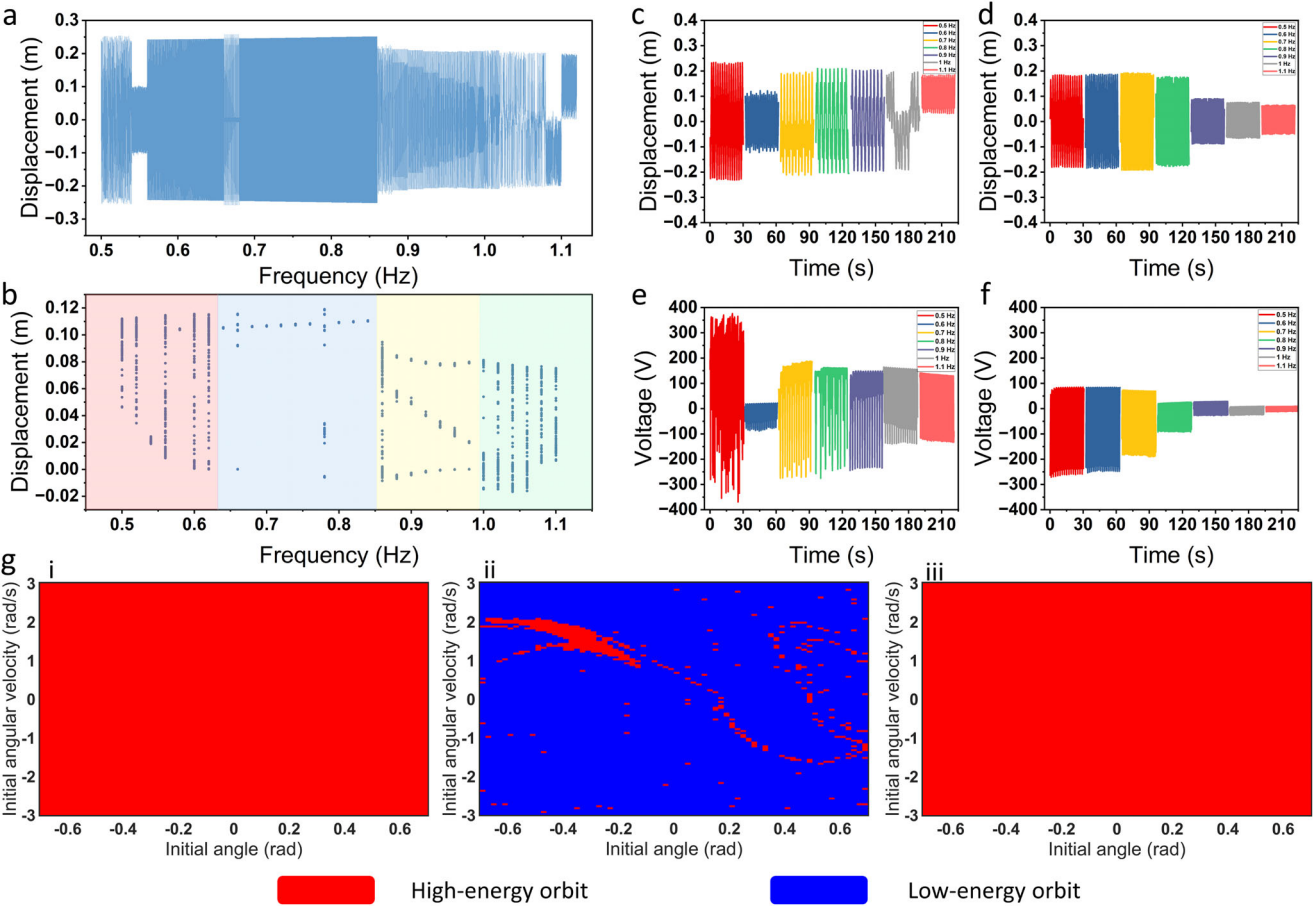
a) The displacement time history response of the motion axis within the frequency range of 0.5 to 1.1 Hz at intervals of 0.02 Hz. b) The bifurcation diagram of the BM‐TENG in the frequency range of 0.5 to 1.1 Hz at intervals of 0.02 Hz. Experimental results of the displacement of the moving shaft in (c) bistable and (d) monostable systems at frequencies ranging from 0.5 to 1.1 Hz. Experimental results of the open‐circuit voltage of e) bistable and f) monostable systems at frequencies ranging from 0.5 Hz to 1.1 Hz. g) The basin attraction diagrams of the BM‐TENG at i) 0.5 Hz, ii) 0.54 Hz, and iii) 0.6 Hz.

For a bistable system, excitation levels, initial parameters and stable points of the system are the most important indicators determining the system dynamic responses. Therefore, it is crucial to analyze the impact of variations in these indicators on the system. This section provides a detailed analysis of the excitation amplitude and frequency, as shown in **Figures** **3** and **4**, which demonstrates the dynamic characteristics of the BM‐TENG under sinusoidal excitation *F*_*e*_ = *A*cos (ϕ*t*). The excitation amplitudes *A* are 19 N, 24 N, 25 N, and 29 N (acceleration about 0.06 g, 0.1 g, 0.11 g, 0.15 g), the excitation ϕ frequency is 0.6 Hz. The other parameters are shown in **Table** **1**. Under the constant excitation frequency, as the excitation amplitude increases, the system transitions through different motions: intra‐well motion, period‐doubling motion, chaotic motion and inter‐well motion.

**Table 1:** Parameters and Values of the BM‐TENG.

**Parameter**	**Value**
Electrode layers *n*	3
Thickness of PTFE membrane *d*	130 µm
Relative permittivity of PTFE membrane ϵ_*r*_	2.2
Surface tribo‐charge density σ	5 µC/^2^
Vacuum permittivity ϵ_0_	8.854 × 10^−12^ F/m
Length of PTFE membrane *l*	0.115 m
Width of PTFE membrane *w*_1_	0.098 m
Spring Stiffness *k*_1_	520 N/m
Mass of mover(Load mass) *m*_1_	11 kg
Friction between electrodes *f*_*f*_	4 N
Friction between motor and mover mass *F*_*m*_	13 N

When the system moves under sinusoidal excitation, the simulation results of the phase diagram (blue line), Poincaré map (red dots), the time histories of displacement, and the Fast Fourier Transform are shown in Figure 3a–d. When the excitation amplitude *A* = 19 N, the system exhibits intra‐well motion. As shown in Figure 3a‐I, the Poincaré map, consisting of red dots, shows that all the red points converge to a single stable location, indicating that the motion of the moving shaft is periodic. Figure 3a‐II further confirms that the moving shaft continuously oscillates around this stable location with a small amplitude. The Fast Fourier Transform of the displacement curve shows two prominent peaks: one at 0.6 Hz, which corresponds to the inherent frequency of the input excitation, and another at 0 Hz. This is because the moving shaft vibrates near one of the stable points which causes the motion trajectory of the system to be biased. The bias of the system motion is manifested as the presence of a 0 Hz frequency component in the Fast Fourier Transform.

When the excitation amplitude *A* = 24 N, the system exhibits period‐doubling motion. Although the system motion trajectory becomes larger, the phase diagram of the system still rotates around two stable points. As the excitation amplitude increases, the system has enough energy to escape the attraction of one stable point, causing the displacement of the moving shaft to increase. The Poincare diagram shows that the red points converge and are distributed at three points on the curve of the phase diagram, indicating that the system's period has become three times the original period. As shown in Figure 3b‐II, the displacement period of the moving shaft is three times the excitation frequency. Furthermore, in the Fast Fourier Transform, apart from the inherent frequency of 0.6 Hz, a peak appears at 0.2 Hz, which is exactly one‐third of the inherent frequency.

When the excitation amplitude is set to *A* = 25 N, the system exhibits evident chaotic behavior. Figure 3c‐I shows a densely filled phase trajectory and a scattered Poincaré map with hierarchical patterns, which indicates the chaotic behavior. In Figure 3c‐II, the moving shaft displacement is highly irregular with random bidirectional oscillations. The frequency spectrum reveals uniform low frequency components (0–0.6 Hz), further confirming chaotic dynamics. These observations confirm that the system undergoes chaotic motion under this excitation condition. Furthermore, given the deterministic mapping between the system's mechanical motion and the output voltage (as expressed in Equation (8)), the chaotic dynamics imply that the output voltage fluctuates irregularly between high and low values.

When the excitation amplitude *A* = 29 N, the system enters inter‐well motion. After the system stabilizes, the phase diagram in Figure 3d‐I appears quite simple, with the system's angle and angular velocity staying near large values. The points in the Poincaré map converge again. Figure 3d‐II shows that the displacement amplitude of the moving shaft is very large and changes rapidly, indicating a high velocity of the moving shaft. Inter‐well motion is the most ideal state of motion, when the moving shaft has the greatest displacement and speed, resulting in the peak voltage and current. The Fast Fourier Transform shows a small peak at 1.8 Hz. This is due to the system's nonlinearity, which causes the system to generate a third‐order harmonic with lower energy.

Figure 3e presents experimental results of the system's phase diagram for excitation amplitudes of 19, 23, 26, and 29 N, where the horizontal and vertical axes represent the displacement and velocity of the moving shaft, respectively. The red and green lines represent periodic intra‐well and inter‐well motion, respectively, while the blue lines, which circle around two stable points, indicate period‐doubling motion. The yellow line appears irregular, indicating chaotic motion. In fact, due to inaccuracies in the measurement of the friction force, the actual friction force slightly deviates from the simulation, resulting in slight differences between the forces required for the system's four different types of motion and those in the simulation. However, the overall trend remains consistent. Meanwhile, as observed in Figure 3a, b, c and d‐II, the experimental results of the displacement of the moving shaft align well with the simulation in terms of general trends.

Figure 3f,g shows the time histories of the open‐circuit voltage and the short‐circuit current of the BM‐TENG under four different motions. These figures reveal the mapping relationship between the displacement of the moving shaft and the electrical performance of the system. In Figure 3f, the open‐circuit voltage increases as the excitation amplitude increases. The voltage rises sharply when the oscillating electrode is about to separate from the fixed electrode, while the voltage increases slowly when the electrodes first begin to separate. Therefore, when the displacement of the moving shaft is small, the open‐circuit voltage is low. Conversely, when the moving shaft approaches the limit of the structure, the open‐circuit voltage becomes large. In fact, the change in excitation amplitude is manifested as the displacement amplitude and velocity of the moving shaft. When the displacement amplitude of the moving shaft is larger, the electrical output performance of the system improves. Therefore, studying the characteristics of bistable structures holds the key to exploring the output performance of BM‐TENG.

Figure 4a presents the displacement curve of the moving shaft in the BM‐TENG with the frequency sweep simulation at excitation frequencies ranging from 0.5 to 1.1 Hz under 30 N. It is clearly observed that at low frequencies (0.5 to 0.85 Hz), the moving shaft has enough energy to overcome the potential barrier, exhibiting a larger vibration amplitude. As the excitation frequency increases from 0.85 to 1.05 Hz, the vibration amplitude of the moving shaft suddenly decreases. When the frequency continues to increase to 1.1 Hz, the moving shaft does not have enough energy to cross the potential barrier, resulting in a smaller amplitude.

Figure 4b shows the bifurcation diagram of the system at excitation frequencies from 0.5 to 1.1 Hz, which facilitates a deeper analysis of the motion characteristics of the system at different excitation frequencies. Overall, Figure 4b can be divided into four sections. In the low and high frequency regions of the diagram (indicated by red and green backgrounds), the points on the bifurcation diagram form a continuous line at each frequency, indicating that the motions of the system at these frequencies are irregular, exhibiting chaotic behavior. In the blue background region, the points concentrate around a single point at each frequency, implying that the system exhibits periodic motion. Similarly, when the points are concentrated around three points at each frequency, corresponding to a triple‐period motion. Comparing the vertical coordinates of points at different frequencies has no practical significance. Observing the distribution at each frequency point can help analyze the system motion state. In fact, in Figure 4a,b, it can be concluded that the higher the excitation frequency, the less energy the system obtains per cycle. And there is always chaotic motion in the process of each stage transformation.

In the BM‐TENG, the selection of the spring's natural length directly affects the analysis of the system's kinematic characteristics. Defined *x*_0_ as the separation distance between the electrodes when the two springs are at their natural lengths. Due to the symmetry of the structure, as long as *x*_0_ ≠ 0, the system will always have two stable points. Therefore, when selecting the natural length of the spring, there is a special case distinct from others, where the natural length is chosen at *x*_0_ = 0 m. In this case, the system is referred to as a monostable system. Thus, it is necessary to analyze the kinematic characteristics of both monostable and bistable systems under the same conditions. Figure 4c,d shows the experimental results of the displacement time histories for the bistable system (*x*_0_ = 0.035 m) and the monostable system (*x*_0_ = 0 m) under excitation of 30 N, respectively. It can be observed that the displacement of the moving shaft within bistable system is always larger than that in monostable system across all low‐frequency ranges except around 0.6 Hz. The monostable system appears clearly, the system has only one kind of motion. When the frequency is between 0.5 and 0.8 Hz, the displacement amplitude of the monostable system is large. However, as the frequency increases, the displacement amplitude suddenly decreases. Near 0.9 Hz, the moving shaft in the bistable system still exhibits inter‐well motion. This indicates that the bistable mechanism is not only more suitable for low‐frequency excitation, but widens the operational frequency band of the system compared with monostable system. Figure 4e and f show the time histories of open‐circuit voltage for both systems. It is evident that the open‐circuit voltage in the bistable system is significantly higher than that in the monostable system. The peak‐to‐peak voltage of bistable system reaches 730 V under 0.5 Hz. Moreover, when the moving shaft moves to both sides of the structure, the open‐circuit voltage increases rapidly. Therefore, although the displacement amplitudes of both systems are similar near 1.1 Hz, the moving shaft in the bistable system moves near one of its stable points, which is closer to one side of the structure. In contrast, the stable point of the monostable system is at the center of the structure. This results in the bistable system having a higher open‐circuit voltage.

A particular observation appears in Figure 4a,c, e, where a strange frequency point is observed. Both the simulation and experimental results show that, near 0.6 Hz, the bistable system has a very small displacement, even smaller than that of the monostable system under the same excitation condition. This is because the bistable mechanism is exceptionally complex. Under certain excitation conditions, the system may exhibit two different motion trajectories within a narrow frequency band: high‐energy orbit and low‐energy orbit. In fact, the initial conditions (initial angle θ and initial angular velocity ω) are the key factors that determine whether the system will settle on the high‐energy or low‐energy orbit. Figure 4g shows the attractor simulation for the bistable system at 0.5, 0.54, and 0.6 Hz. When the excitation frequency is 0.54 Hz, the system exhibits two trajectories. At 0.5 Hz or 0.6 Hz, regardless of initial conditions, the system always locates in the high‐energy orbit. Thereby, it can be inferred that when the system has two distinct energy levels, changing the initial conditions can alter the motion trajectory of the system to high‐energy orbit. Additionally, applying a suitable perturbation can also enable the system to transition from the low‐energy orbit to the high‐energy orbit.

### 2.4 Parametric Analyses of the BM‐TENG

The previous section provided a detailed analysis of the excitation and initial conditions of the system. However, the essence of the motion in the BM‐TENG system is mostly determined by the characteristics of the spring assembly. This section will delve deeper into the inherent parameters of the spring assembly and offer theoretical recommendations on how to choose springs under a certain excitation level.

**Figure 5:**
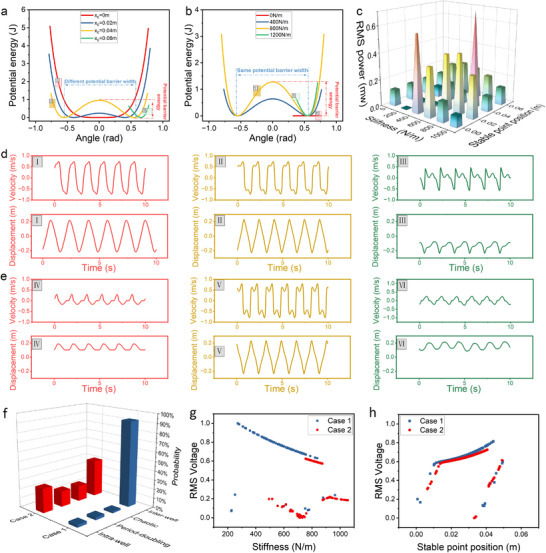
Dynamic analyses of the BM‐TENG with different parameters. a) Potential energy of the BM‐TENG at different stable points. b) Potential energy of the BM‐TENG for varying spring stiffness. c) Output performance of the BM‐TENG at different spring stiffness and stable points. d) The displacement and velocity of the moving shaft corresponding to the three different stable points in Figure 5a. e) The displacement and velocity of the moving shaft corresponding to the three different spring stiffness in Figure 5b. f) The inter‐well failure possibility of BM‐TENG under excitation amplitudes of 25 N (case 2) and 27 N (case 1) respectively when the spring stiffness changes. g) The normalized RMS voltages of BM‐TENG under excitation amplitudes of 25 and 27 N, respectively when the spring stiffness changes. h) The normalized RMS voltages of BM‐TENG under excitation amplitudes of 25 and 27 N, respectively when the stable point position changes.

**Figure** **5**a,b show the potential energy of the system with varying stable points and spring stiffness. In Figure 5a, when *x*_0_ = 0 m, representing a monostable system. As the stable point moves closer to the two sides of the structure, both the width and height of the potential barrier increase, requiring more energy for the system to cross the barrier. Until *x*_0_ = 0.06 m, the system lacks sufficient energy to cross the potential barrier and then transitions from the inter‐well motion to the intra‐well motion, with the moving shaft vibrating near a single stable point. In Figure 5b, when *k* = 0 Nm^−1^, the system has no spring, and the total potential energy of the system is independent of the position of the moving shaft and is always zero. At this time, the length of potential energy curve is very short, indicating that the moving shaft vibrates within a small range. As the spring stiffness increases, the potential barrier gradually increases until the system again lacks sufficient energy to be attracted to a stable point.

Figure 5d shows the displacement and velocity time histories of the moving shaft for the different stable points in Figure 5a. The displacement and velocity of the moving shaft in bistable system (subplot II) are slightly larger than in monostable system (subplot I). Section 2.3 has indicated that as the stable points move closer to the sides of the structure, the voltage rises more quickly. Theoretically, setting the stable points closer to the sides would increase the output performance of the system. However, in practice, as the stable points move closer to the ends, the width and height of the potential barrier increase, requiring more energy to cross the barrier. If the system does not have enough energy, the moving shaft will be attracted to one of the stable points, resulting in smaller displacement and velocity, as shown in subplot III. Figure 5e illustrates the displacement and velocity time histories of the moving shaft for different spring stiffness. When there is no spring, due to friction and load mass, the excitation level is not sufficient to cause significant displacement and velocity of the moving shaft as shown in subplot IV. When the stiffness of the springs is 400 Nm^−1^ (subplot V), the bistable system exhibits inter‐well motion. However, if the spring stiffness is too large, the system has insufficient energy to cross the energy barrier, and the moving shaft will be attracted to one of the stable points (subplot VI).

Figure 5c shows the influence of different spring stiffness and stable point positions on the system output performance. The figure clearly illustrates that when the system does not have springs, the output power is very small. The monostable system performs high output when the spring stiffness is around 500 Nm^−1^. However, as the spring stiffness increases further, the moving shaft is attracted to the central stable point, and the displacement amplitude decreases, leading to reduced output performance. In Figure 5c, the bistable system maintains high output over a certain range, as the spring stiffness and stable point selection result in a similar height of the potential barrier.

Based on the above analysis, the bistable system can exhibit the best electrical performance during periodic inter‐well motion. Under certain excitation, adjusting the parameters of the spring assembly can alter the energy required to cross the barrier. However, as mentioned in Section 2.3, there is a transitional period of chaos between inter‐well and intra‐well motion. Thus, when determining the spring parameters, it is crucial to ensure that the barrier energy is slightly smaller than the energy of the system, which will guarantee periodic inter‐well motion in a bistable system thereby achieving high‐performance output. It is worth mentioning that, if the energy of the system is high enough, even monostable system and system without spring will exhibit higher output power. In that case, the number of electrode plates can be increased to increase the friction area and reduce the energy of the system. At this time, the introduction of bistable mechanism not only ensures more friction area but also achieves ideal kinematic characteristics. Therefore, the bistable mechanism plays a crucial role in improving the output performance of the system. When external excitation is small, the bistable system can also perform better dynamic characteristics, thereby enhancing the overall energy conversion efficiency.

In Figure 5f,g,a Monte Carlo simulation to examine in detail how variations in spring stiffness influence the failure rate of inter‐well motion is conducted. Two different excitation scenarios were simulated: one with an excitation amplitude of 27 N (Case 1), and another with 25 N (Case 2). Based on extensive simulations, we observed that inter‐well motion typically occurs near spring stiffness values of 600 and 800 Nm^−1^ under the respective excitation amplitudes. Therefore, set the mean spring stiffness at 600 and 800 Nm^−1^ accordingly. Considering possible fluctuations in real world environments, assume that the spring stiffness follows a normal distribution within the ranges of 300–900 Nm^−1^ (for 27 N) and 500–1100 Nm^−1^ (for 25 N). For the 27 N excitation, the probabilities of intra‐well, period‐doubling, chaotic, and inter‐well motion were found to be 3%, 3%, 2%, and 92%, respectively. In contrast, for the 25 N excitation, these probabilities were 26%, 17%, 17%, and 40%. These results indicate that although environmental corrosion or mechanical wear may lead to significant variations in spring stiffness, the system still demonstrates strong robustness under higher excitation amplitudes, with inter‐well motion occurring in more than 90% of simulations. Under lower excitation amplitudes, however, the system becomes more sensitive to changes in spring stiffness, suggesting that lower excitation amplitudes require more precise parameter matching.

Figure 5h shows the system sensitivity to small variations in the equilibrium position. For both excitation cases (27 and 25 N), the spring stiffness was fixed at 800 Nm^−1^ and the mean equilibrium position was set at 0.03 m. As shown in Figure 5h, the system sensitivity to equilibrium position shifts is similar across different excitation amplitudes. However, a higher excitation amplitude enables a wider range of inter‐well motion. Compared to Case 2, the transition point from inter‐well motion to period‐doubling or chaotic motion in Case 1 shifts backward by approximately 0.004 m. It can be observed that when the equilibrium position lies within the range of 0.01–0.044 m (for 27 N) and 0.01–0.04 m (for 25 N), the bistable system demonstrates high‐output inter‐well motion. In contrast, when the equilibrium position is close to the center (i.e., 0–0.01 m), the system tends to behave as monostable, with a significant drop in output voltage. Under both conditions, when the equilibrium position exceeds 0.044 m (for 27 N) or 0.04 m (for 25 N), the system begins to exhibit quasi‐periodic, chaotic, or intra‐well motion, leading to a reduction in RMS output voltage. Nevertheless, despite the presence of multiple motion states, the bistable system still produces a higher RMS voltage in most cases compared to the monostable system.

### 2.5 Output Performances of the BM‐TENG

**Figure 6:**
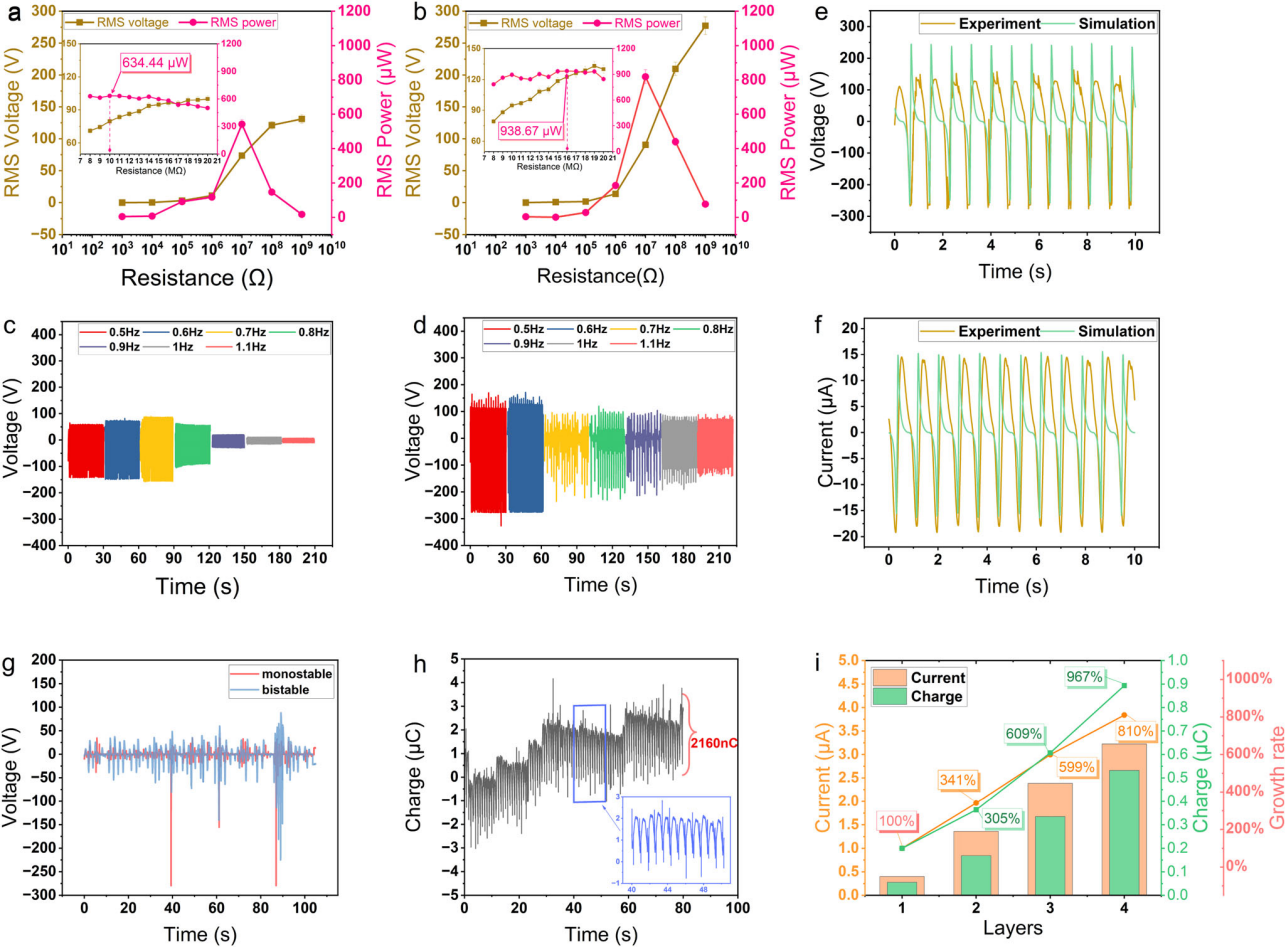
RMS voltage and power of the a) bistable system and b) monostable system at different loads. c) Output voltage of the monostable system at different frequencies with a 10 MΩ load. d) Output voltage of the bistable system at different frequencies with a 16 MΩ load. e) Output voltage and f) current of the BM‐TENG under 0.6 Hz frequency and 31 N excitation with a 16 MΩ load. g) Output voltage of monostable and bistable systems under random excitation. h) Charge transfer of the BM‐TENG. i) Effect of the number of layers in the BM‐TENG on its output performance.

**Figure** **6**a,b displays the experimental results of impedance matching for the monostable and bistable systems. When the excitation amplitude is 31 N (acceleration about 0.18 g) and the excitation frequency is 0.6 Hz, the monostable system has a maximum root mean square (RMS) power of 634.44 µW with an optimal external resistance of 10 MΩ, while the bistable system has a maximum RMS power of 938.67 µW with an optimal external resistance of 16 MΩ. This indicates a 48% increase of power in the bistable system compared with the monostable one. When the circuit is close to the open circuit, the RMS voltage monostable system is about 127 V, and increased to about 281 V by 121% in the bistable system. Meanwhile, the instantaneous power of the bistable system can calculate to be 5.08 mW, as the maximum voltage shown in Figure 6d. The time histories of output voltage under the optimal resistance for different excitation frequencies in the monostable and bistable systems are shown in Figure 6c,d, respectively. The output voltage amplitude of the monostable system initially increases with frequency but sharply drops when the excitation frequency exceeds 0.9 Hz. In this case, the moving shaft oscillates with a very small displacement at the center of the structure. The bistable system, under excitation at 31 N and 0.6 Hz, does not exhibit the low‐energy orbit shown in Section 2.3, so the system maintains periodic inter‐well motion until 0.6 Hz. After 0.6 Hz, the system experiences period‐doubling and chaotic motion. Due to the stable points are located closer to both ends of the structure, the moving shaft motion is closer to the ends because of the attraction of the stable points which causes the output voltage amplitude remains relatively high. Even when the system exhibits intra‐well motion, the output voltage does not exhibit a noticeable downward trend. This is because the moving shaft in bistable system exhibits periodic vibration around one of the stable points which has higher separation distance compared with monostable system. This demonstrates the another advantage of the bistable mechanism in increasing the system output voltage.

Figure 6e,f shows the experimental and simulation results of the output voltage and current under periodic inter‐well motion in the bistable system. In Figure 6e, the experimental and simulated output voltages match very well during negative values but show some discrepancy during positive values. This phenomenon is mainly due to two reasons. First, when the oscillating and fixed electrodes are separated, the potential difference increases, while when they come into contact, the potential difference decreases. This results in different voltages during contact and separation. Second, each time the moving shaft hits the limit device, inelastic collisions occur so that the system loses some kinetic energy. During each cycle, the speed at which the oscillating electrode and the fixed electrode come into contact is slightly lower than the speed at which they separate, resulting in a decrease in voltage. Furthermore, the voltage and current curves in the experiment are evenly distributed, rather than having high peaks but short durations in each cycle, which is one of the reasons why the BM‐TENG can output high performance.

Figure 6g shows the output voltage of the monostable and bistable systems under the same small random excitation (both loaded with a 20 MΩ resistor), where the blue line almost covers the red line. During the entire 100‐second working period, since the excitation is identical, the overall output voltage trends of both systems are completely consistent, which indicates the bistable system still has an advantage over the monostable system under random excitations. This is because the stable points are closer to the two sides in bistable system, leading to higher displacement of the moving shaft. Besides, the bistable system basically keeps inter‐well motion to achieve greater displacement especially around 90 s. Figure 6h shows the charge accumulation of the bistable system during periodic inter‐well motion, with the peak‐to‐peak value of the charge remaining at approximately 2.16 µC. When measuring the charge with the Keithley 6514 electrometer, due to the internal structure of the electrometer, there is a slight deviation, and the overall trend of the measured charge always shifts to one side. However, this shift does not affect the measurement of the value of the charge. Figure 6i, depicting the experimental results with different numbers of BM‐TENG layers, shows that the peak‐to‐peak values of charge and current increase as the layer number increases. The overall growth curve of the current and charge is approximately linear, indicating that when the number of layers increases to *n* layers, the output power of BM‐TENG will be *n* times that of a single layer. It is worth noting that the more layers, the greater the pressure between the oscillating and fixed electrodes, resulting in more charge transfer due to friction. When the BM‐TENG has only one layer, the pressure between the electrode plates is small, and the output performance is not ideal. This explains why the output performance of two layers is 341% better than that of a single layer. Therefore, adding more layers allows the bottom electrode plate to carry more charge, leading to higher output power while keeping the volume of nanogenerator nearly unchanged. This indicates that with the optimization, the power density of the proposed nanogenerator can be further increased.

### 2.6 Application tests for Wireless Sensing and Warning Lights

As shown in Figure 1, the demand for ocean‐based Internet of Things is growing. Monitoring the chemical composition and physical state of the ocean is very important for the smooth running of the oil drilling platform. In addition, due to the uncertainty of the marine environment, it is also crucial to set up a cordon along the shore to prevent people from straying into dangerous areas. Therefore, relevant experiments are conducted in this chapter to verify the feasibility of practical application of BM‐TENG.

**Figure 7:**
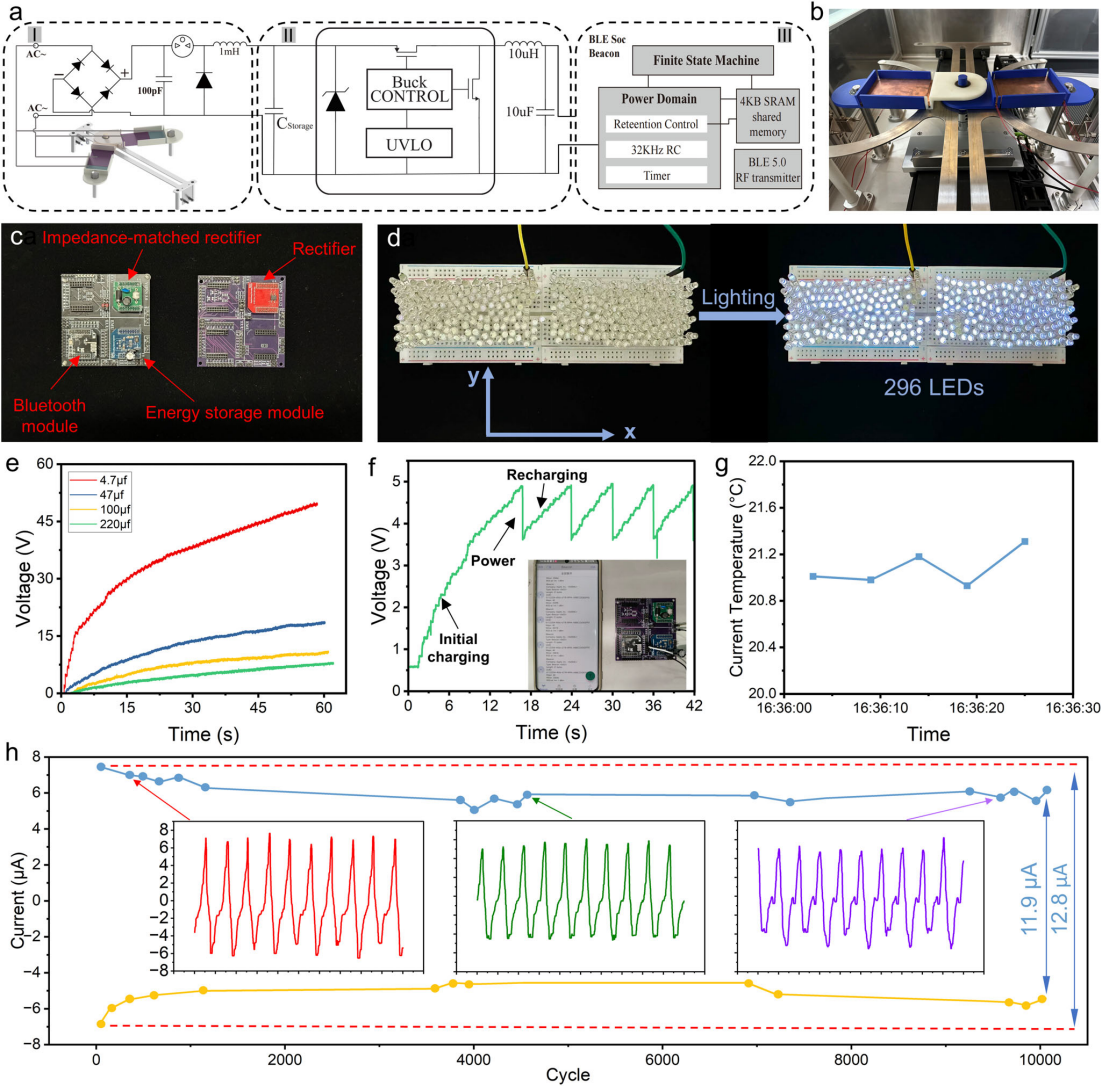
Demonstrations of the application of the BM‐TENG. a) Circuit diagram of BM‐TENG driving Bluetooth module for temperature detection. b) Actual picture of BM‐TENG on linear motor. c) Energy storage integrated board and rectifier bridge. d) The real time lighting photo of 296 light emitting diodes (LEDs) driven by BM‐TENG. e) Charging rate for different capacitors. f) BM‐TENG supplies power for Bluetooth module integrated with temperature sensor. g) Real time temperature of the sensor. h) Durability test of BM‐TENG under 10000 cycles.

Through extensive theoretical predictions and experimental validations, the BM‐TENG demonstrates sufficient output performance to power small electronic devices. This study is conducted at an excitation frequency of 0.6 Hz, an amplitude of 31 N (acceleration about 0.18 g), 3 layers of electrode plates, a spring stiffness of 520 Nm^−1^, and stable points position of *x*_0_ = 0.035 m. In **Figure** **7**a‐I, the 100 pF capacitor serves as the direct load for the energy harvester. The gas discharge tube acts as a switch, allowing the capacitor to accumulate sufficient energy under low‐frequency excitation and then rapidly release it upon breakdown of the gas discharge tube. This energy is transferred through a 1 mH inductor into the storage capacitor in the second part. Meanwhile, the voltage across the 100 pF capacitor drops, and the gas discharge tube returns to its cutoff state. Compared to directly connecting the storage capacitor, this circuit configuration provides a higher input impedance for the energy harvester. Figure 7a‐II shows is energy storage module, LTC3588 is used as the core energy management controller and offers significant advantages in energy harvesting. Its design includes two key technical features: firstly, it precisely locks the threshold voltage of the energy storage capacitor at 5 V through an under‐voltage lockout (UVLO) mechanism, effectively preventing energy loss due to over‐discharge; secondly, when the storage voltage reaches the preset threshold, its built‐in synchronous buck converter efficiently converts the 4.9 V input into a 3.3 V DC output, with a conversion efficiency of over 94%. These features make LTC3588 an ideal choice for self‐powered IoT systems, ensuring both the safety of energy storage and the efficient distribution and utilization of power. Figure 7b shows the BM‐TENG mounted on a linear motor. The detailed photos of components are given in the Figure S2 (Supporting Information).

The left side of Figure 7c integrates the impedance‐matched rectifier bridge, energy storage module, and Bluetooth module, while the right side shows a typical rectifier circuit. In Figure 7d and Movie S1 (Supporting Information), 296 light‐emitting diodes (LEDs) are simultaneously lit by the BM‐TENG with the rectifier bridge. Within one cycle, the LEDs exhibit two brightness peaks. This feature better aligns with the lighting requirements for maritime warning lines. Notably, the LEDs are connected in series along the x‐direction and in parallel along the y‐direction.

Figure 7e illustrates the rate of voltage rise when the voltage of the BM‐TENG is charged to different capacitances through an impedance‐matched rectifier bridge. After continuous charging for 60 s, the voltages of capacitors with 4.7 µF, 47, 100, and 220 µF reach 50, 18.7, 11.1, and 8.1 V, respectively. Figure 7f and Movie S2 (Supporting Information) show the voltage variation of the capacitor (220 µF) in the energy storage module when the BM‐TENG is used to power a Bluetooth module with a temperature sensor. Initially, the BM‐TENG charges the energy storage capacitor, and after continuous charging for 18 seconds, the voltage of the storage capacitor reaches 4.9 V. The capacitor then begins to discharge to supply power to the Bluetooth module, causing its voltage to suddenly drop to 3.3 V. Subsequently, the voltage of the energy storage capacitor is recharged to 4.9 V within the next 5 s, preparing it for the next power supply cycle to the Bluetooth module. Specifically, the several voltage drops correspond to the five times the phone receives Bluetooth transmission signals (Figure 7f).

In Figure 7f, there will be a Minor value every time the signal appears so that the signal can display the current environmental temperature, which can be obtained by a simple formula calculation as follows:

Current Temperature (*CT*) = Minor // 256 * 0.01 + Minor % 256 * 2.

In Figure 7f, Minor = 25866, and the temperature is calculated as:

*CT* = (25866 // 256) * 0.01 + (25866 % 256) * 2 = 21.01 °where // is the division operation but omits the decimal part and does not round, % is the remainder operation. Therefore, the current environmental temperature is calculated to be 22.04 °.

Figure 7g shows five temperature signals from the sensor in 25 s. The current temperature is about 21 °. This demonstrates that the BM‐TENG is fully capable of providing real‐time power to the Bluetooth module. The actual energy consumption of the Bluetooth module *E*_*c*_ and the output power for Bluetooth module of BM‐TENG *P*_*c*_ can be calculated as:

(9) $$E_{c}=0.5C_{sto}\left(V_{af}^{2}-V_{bf}^{2}\right),\;P_{c}=E_{c}/T$$

where, in this section, *C*_*sto*_ is capacitor capacity is 220 µF, *V*_*af*_ is the maximum voltage before voltage drops is 4.9 V and *V*_*bf*_ is the minimum voltage after voltage drops is 3.3 V. For the experimental condition in this section, *E*_*c*_ can calculate to be 1.4432 J, *P*_*c*_ can calculate to be 0.289 mW. Therefore, the actual power of the BM‐TENG to drive the Bluetooth module after it is rectified by impedance‐matched circuit accounts for 30.7% of the optimal RMS power.

As shown in Figure 7h, a 10000‐cycle stability test of the BM‐TENG in a high‐salt (3.5%), high‐humidity (74%) environment is conducted. During the initial 1000 cycles, the output current exhibited a slight decline. After this stage, the output current gradually stabilized. Upon completion of 10000 cycles, the output current had decreased by approximately 7% compared to its initial value. These findings suggest that the BM‐TENG possesses good stability under harsh marine conditions. Moreover, considering the practical application of the BM‐TENG in marine environments, the glass or acrylic sheets can be employed to fully seal the device, effectively preventing the ingress of water or moisture into the device.

Additionally, one of the key advantages of the BM‐TENG proposed in this work is its ability to increase the number of triboelectric units through a vertically stacked design, thereby achieving an enhancement in power output without significantly increasing the device volume (see Figure 6i). In the current experiments, the reported RMS power output of 0.94 mW and the maximum output power of 5.04 mW were obtained using a structure consisting of only three layers on each side (a total of six layers). Notably, the total power output and power density can be further improved by increasing the number of layers. With an increasing number of layers, the power density of the BM‐TENG can reach up to 32.96 W/m^3^.

**Table** **2** summarizes the power densities reported in previous studies on TENGs. It can be observed that the proposed device exhibits superior power density under the obviously ultra‐low excitation frequency. It is worth noting that the current oscillating electrodes are made of 1 mm thick copper plates. In fact, the required structural stiffness for sustaining system stability can still be fully satisfied by reducing the thickness to 0.3 mm or even lower. Using thinner metal plates can significantly reduce the thickness of a single layer, thereby increasing the power density. This could help narrow the “order‐of‐magnitude gap” between the device output and the vast potential of ocean energy.

**Table 2:** The comparison of power density between BM‐TENG and the studies of other scholars (contact‐separation (CS) mode, plane‐sliding (PS) mode, and freestanding dielectric‐layer (FD) mode).

Device	Mode	Test	Power density	Normalized power density	Refs.
This work	PS	Linear motor 0.6 Hz	32.96 Wm^−3^	54.9 Wm^−3^ · Hz^−1^	—
Tower‐like TENG	FD	—	10.6 Wm^−3^	—	[61]
Dense point contacts TENG	FD	Linear motor 0.8 Hz	20.57 Wm^−3^	25.7 Wm^−3^ · Hz^−1^	[62]
Spring‐assisted TENG	CS	Linear motor 1.86 Hz	1.84 Wm^−3^	0.99 Wm^−3^ · Hz^−1^	[63]
Spring‐assist multilayered TENG	CS	Water tank 1 Hz	15.3 Wm^−3^	15.3 Wm^−3^ · Hz^−1^	[64]
Water‐tube‐based TENG	FD	Linear motor 1 Hz	13.1 Wm^−3^	13.1 Wm^−3^ · Hz^−1^	[65]
Snake‐like TENG	FD	Water tank 0.5 Hz	3 Wm^−3^	6 Wm^−3^ · Hz^−1^	[66]
Open book‐like TENG	CS	Linear motor 1 Hz	9.675 Wm^−3^	9.675 Wm^−3^ · Hz^−1^	[67]
Stacked pendulum TENG	FD	Water tank 0.3 Hz	14.71 Wm^−3^	49.1 Wm^−3^ · Hz^−1^	[48]

## 3 Conclusion

In conclusion, a novel and versatile multi‐layer TENG based on the bistable dynamic mechanism to boost energy conversion efficiency was proposed. The structure is capable of converting the impact force of ocean waves or the gravitational potential energy generated by the oscillations of buoy into reciprocating motion of the moving shaft within the structure. Based on rigorous simulations and experimental investigations, this study demonstrates that the BM‐TENG can effectively address the issue of low energy output in traditional TENGs under ultra‐low‐frequency and low‐amplitude ocean wave excitations. In addition, the bistable mechanism broadens the operational frequency band compared with the monostable system. Besides, since the stable points are located close to both sides, the displacement of the system during intra‐well motion remains high. Through extensive dynamic analysis, the influence of external excitations and the spring characteristics on the system's motion behavior is explored. Under the same weak input conditions, the bistable system exhibits higher energy conversion efficiency than the monostable and linear systems, no matter in intra‐well or inter‐well motion. When the BM‐TENG is in periodic inter‐well movement, its RMS power can reach 0.94 mW, which is 48% higher than that of the monostable system under 0.6 Hz. Meanwhile, the BM‐TENG achieves an instantaneous maximum power of 5 mW, a peak‐to‐peak voltage of 730 V, and a transferred charge of 2.16 µC. Based on the theoretical analysis, the BM‐TENG can efficiently convert external vibrational energy into electrical energy, successfully powering lighting system and Bluetooth temperature sensor.

Additionally, due to the redundancy inherent in the multi‐layer parallel mechanism of the structure, the overall system can maintain normal operation even if some of the friction units fail, which is especially important in complex marine environments. More importantly, the structural design of the BM‐TENG offers good modularity and scalability. It can be deployed not only on nearshore buoys, drilling platforms, and other marine infrastructure, but also widely in applications involving low‐frequency reciprocating motion, such as bridge trusses, streetlights, and billboards, enabling distributed, low‐power energy harvesting and sensing. With the integration of wireless communication systems, a BM‐TENG‐based “Smart IoT” can be established to monitor and transmit data on parameters of climate and environment. This plays a key role in ocean and infrastructure safety monitoring. This research provides new insights for building the next‐generation marine IoT and smart monitoring systems for infrastructure.

## Conflict of Interest

The authors declare no conflict of interest.

## Supporting information

Supporting Information

Supplemental Movie 1

Supplemental Movie 2

## Data Availability

The data that support the findings of this study are available from the corresponding author upon reasonable request.
