# Supplementary material for: Bistable Multi‐Layer Triboelectric Nanogenerator for Harvesting Random and Ultra‐Low‐Frequency Vibration Energy with Increased Charge Transfer
Source: Adv Sci (Weinh). 2025 Jun 24;12(31):e05246. doi: 10.1002/advs.202505246 (PMC12376591; doi:10.1002/advs.202505246)
Supplement: Supplementary file 1 — Supporting Information [file ADVS-12-e05246-s002.docx]

**Bistable Multi-Layer Triboelectric Nanogenerator for Harvesting Random and Ultra-Low-Frequency Vibration Energy with Increased Charge Transfer**

Yi Guan, Xin Li, Zehan Wei, Mianxin Xiao, Zhihui Lai, Shuxiang Dong, Daniil Yurchenko, Shitong Fang*

**Supporting Information**

**Contents:**

Section S1: Derivation of kinetic and electrical equations of BM-TENG under external excitation.

Section S2: Detailed structure of BM-TENG components.

Section S3: Experimental section.

**This file includes:**

Figure S1. The positional relationship of the components of BM-TENG and the simplified connectors. Figure S2. Detailed demonstration of BM-TENG components and experimental instruments.

**Section S1: Derivation of kinetic and electrical equations of BM-TENG under external excitation.**

**
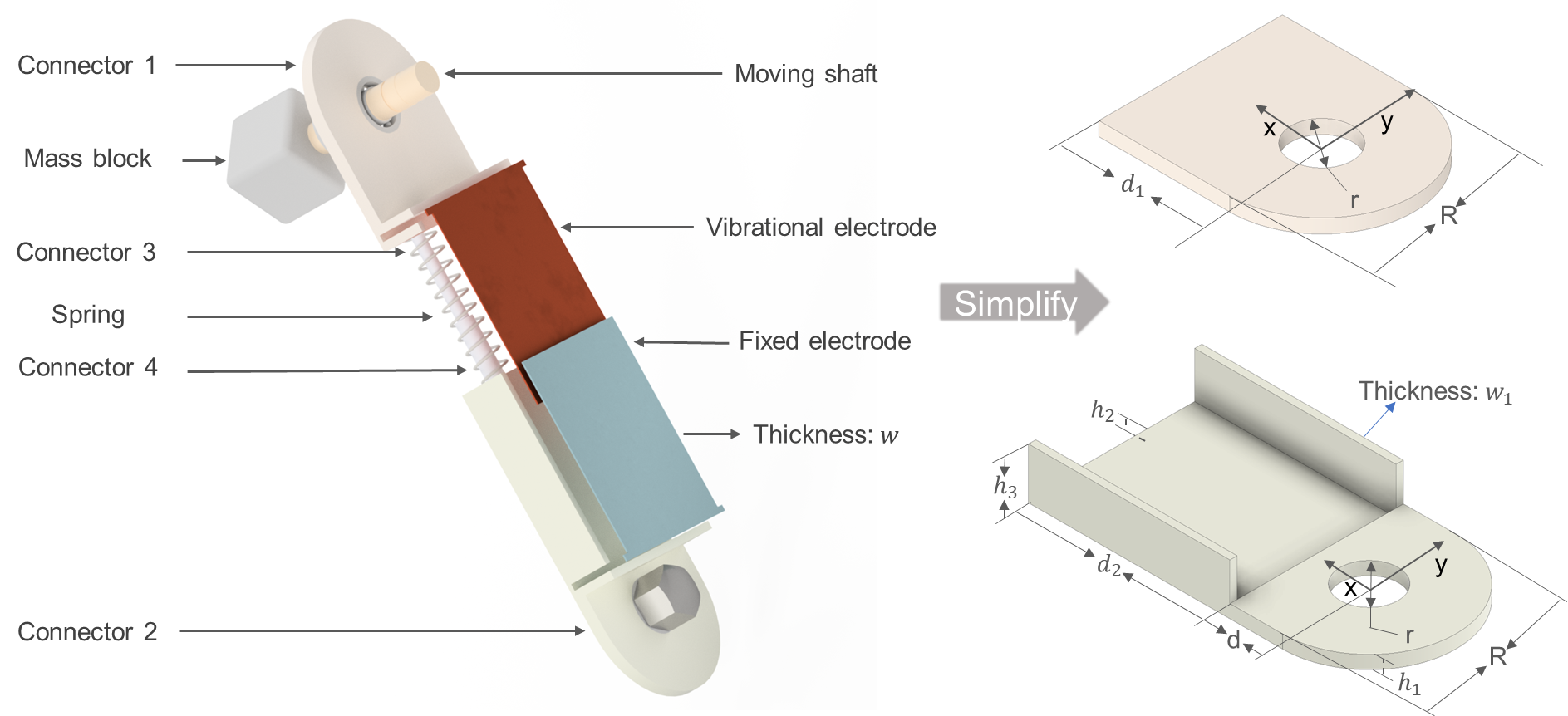
**

Figure S1. The positional relationship of the components of BM-TENG and the simplified connectors.

**S1.1 Dynamic modeling**

In the main text, the establishment of the dynamic model is based on the Lagrange dynamic equation:

$$\begin{aligned} \frac{d}{dt}\left( \frac{\delta L}{\delta\dot{q_{k}}} \right)-\frac{\delta L}{\delta q_{k}}{=Q}_{k}\#\left( s1 \right) \end{aligned}$$

where

$$\begin{aligned} L=E_{k1}+E_{k2}+E_{k3}-E_{p1}-E_{p2}\#\left( s2 \right) \end{aligned}$$

$$\begin{aligned} Q_{k}=Q_{e}+Q_{f}\#\left( s3 \right) \end{aligned}$$

Section S1 will give a detailed derivation of all components potential and kinetic energy within BM-TENG and nonconservative forces $Q_{k}$ mapped on generalized coordinate $q_{k}$.

The moving shaft and fixed electrode have only one degree of freedom: the former moves linearly back and forth along the guiding slot, and the latter rotates around the fixed shaft. Regarding $L_{s}$ as the distance from the center of the moving shaft to the center of fixed shaft when the moving shaft is in the middle position, then the formula is as follows:

$$\begin{aligned} E_{k1}=\frac{1}{2}m_{1}\dot{h}^{2}, E_{k2}=T_{1}+T_{2}=\frac{1}{2}J_{1}\omega^{2}+\frac{1}{2}J_{2}\omega^{2}\#\left( s4 \right) \end{aligned}$$

where

$$\begin{aligned} h=L_{s}\cdot\tan\theta, \dot{h}= \frac{L_{s}\dot{\theta}}{{\cos\theta}^{2}}\#\left( s5 \right) \end{aligned}$$

where $m_{1}$ is the total mass of the moving shaft with bearings attached, h is the distance from the middle point of the slot to the moving shaft, $\dot{h}$ is the velocity of the moving shaft in the guiding groove, $\omega$ is the derivative of the generalized coordinate rotation angle $\theta$, i.e., the angular velocity. $T_{1}$ and $T_{2}$ are the kinetic energies of the fixed electrode plate and connector 2, respectively. $J_{1}$ and $J_{2}$ are the moments of inertia of the fixed electrode plate and connector 2:

$$\begin{aligned} J_{1}=m_{2}R^{2}=\int_{w_{1}-\frac{R}{2}}^{\frac{R}{2}-w_{1}} dy\int_{d}^{d_{2}+d} \rho w\left( \sqrt{x^{2}+y^{2}} \right)^{2}dx\#\left( s6 \right) \end{aligned}$$

where $m_{2}$, $\rho$, $w$ are the mass, density, and thickness of the fixed electrode plate, respectively. The shape of connector 2 can be simplified; the simplified $J_{2}$ is as follows:

$$\begin{aligned} J_{2}=\int_{-\frac{R}{2}}^{\frac{R}{2}} dy\int_{-\sqrt{R^{2}-y^{2}}}^{d} \rho h_{1}\left( \sqrt{x^{2}+y^{2}} \right)^{2}dx-\frac{1}{2}\rho_{1}h_{1}\pi r^{2}\left( R^{2}+r^{2} \right) \\ +\int_{-\frac{R}{2}}^{\frac{R}{2}} dy\int_{d}^{d_{2}+d} \rho_{1}h_{2}\left( \sqrt{x^{2}+y^{2}} \right)^{2}dx \\ +2\int_{\frac{R}{2}-w_{1}}^{\frac{R}{2}} dy\int_{d}^{d_{2}+d} \rho_{1}h_{3}\left( \sqrt{x^{2}+y^{2}} \right)^{2}dx\#\left( s7 \right) \end{aligned}$$

where $h_{1}$, $h_{2}$, $h_{3}$ correspond to the heights of the ring, base plate, and vertical plate in connector 2, $\rho_{1}$ is the material density of the connector, and other parameters are shown in the figure.

The movement of the oscillating electrode involves both translation and rotation. It is worth noting that the derivation of the rotational energy of the oscillating electrode plate and connector 1 is similar to the fixed electrode. However, the oscillating electrode plate has an additional movement towards the fixed shaft, and connector 1 has an additional linear motion along the guide slot. The formula is as follows:

$$\begin{aligned} E_{k3}=\frac{1}{2}J_{3}\omega^{2}+\frac{1}{2}J_{4}\omega^{2}+\frac{1}{2}m_{2}{\dot{x_{s}}}^{2}+\frac{1}{2}m_{3}\dot{h}^{2}\#\left( s8 \right) \end{aligned}$$

where

$$\begin{aligned} J_{3}=\int_{-\frac{R}{2}}^{\frac{R}{2}} dy\int_{-\sqrt{R^{2}-y^{2}}}^{d_{1}} \rho h_{1}\left( \sqrt{x^{2}+y^{2}} \right)^{2}dx-\frac{1}{2}\rho_{1}h_{1}\pi r^{2}\left( R^{2}+r^{2} \right)\#\left( s9 \right) \end{aligned}$$

$$\begin{aligned} J_{4}=m_{2}R^{2}=\int_{w_{1}-\frac{R}{2}}^{\frac{R}{2}-w_{1}} dy\int_{d+x_{s}}^{d_{2}+d+x_{s}} \rho w\left( \sqrt{x^{2}+y^{2}} \right)^{2}dx\#\left( s10 \right) \end{aligned}$$

$$\begin{aligned} x_{s}=L_{s}\left( \frac{1}{\cos\theta}-1 \right), \dot{x_{s}}=\frac{L_{s}\dot{\theta}\sin\theta}{{\cos\theta}^{2}}\#\left( s11 \right) \end{aligned}$$

In the above formula, $J_{3}$ is the moment of inertia of the connector 1, $J_{4}$ is the moment of inertia of the oscillating electrode plate. $x_{s}$ represents the separation distance, which is the non-contact length between the fixed electrode plate and the oscillating electrode plate. Defining $x_{s}=0$ when the moving shaft is in the middle position, which means the fixed electrode plate and the oscillating electrode plate are fully overlapped. When the moving shaft is at either end, $x_{s}$ is maximized, meaning the contact area between the fixed and oscillating electrode plates is minimized.

Since this structure is placed horizontally, gravity does no work during the working process, and the whole potential energy of the system consists only of the elastic potential energy of the springs. The elastic potential energy is divided into two parts: one is the elastic potential energy $E_{p1}$, and the other is the elastic potential energy $E_{p2}$ of the stopper spring added to prevent structural damage when the moving shaft reaches the limit positions. Thus, the former accompanies the entire movement process of the structure, while the latter appears only when the moving shaft moves to the sides. The expressions of $E_{p1}$ and $E_{p2}$ are as follows:

$$\begin{aligned} E_{p1}=\frac{1}{2}k_{1}\left( x_{s}-x_{0} \right)^{2}\#\left( s12 \right) \end{aligned}$$

where, $k_{1}$ is the spring stiffness. $x_{o}$ represents the separation distance between the electrodes when the two springs are at their natural lengths. The above formula indicates that when the springs are stretched or compressed, $x_{s}-x_{o}\neq0$, $E_{p1}>0$.

$$\begin{aligned} E_{p2}=\left\{ \begin{aligned} 0, &x_{s}<x_{stop} \\ \frac{1}{2}k_{2}({x_{s}-x_{stop})}^{2}, &x_{s}\geq x_{stop} \end{aligned} \right.\#\left( s13 \right) \end{aligned}$$

where, $k_{2}$ is the stiffness of the limiting spring, and $x_{stop}$ is the separation distance between the oscillating electrode and fixed electrode when the moving shaft just contacts the limiting spring.

Nonconservative force includes external excitation and friction. It is worth mentioning that, friction is always doing negative work. Therefore, the work done by external excitation and friction can be derived as:

$$\begin{aligned} W_{e}=F_{e}h, W_{f}=F_{f}x, F_{e}=A\cos\left( \phi t \right)\#\left( s14 \right) \end{aligned}$$

where $F_{f}$is a vector, whose direction is always opposite to $\dot{x}$. Therefore, $F_{f}=-\frac{\dot{x}}{\left| \dot{x} \right|}f_{f}$, where $f_{f}$ is the value of $F_{f}$ is a scalar, $-\frac{\dot{x}}{\left| \dot{x} \right|}$ represents the direction of $F_{f}$.

$$\begin{aligned} Q_{k}=Q_{e}+Q_{f}=\frac{\partial W_{e}}{\partial q_{k}}+\frac{\partial W_{f}}{\partial q_{k}}=\frac{F_{e}L_{s}}{{\cos\theta}^{2}}-\frac{f_{f}\dot{\theta}\cdot\sin\theta\cdot L_{s}\sin\theta}{\left| \dot{\theta}\sin\theta\right|{\cos\theta}^{2}}=\frac{F_{e}L_{s}}{{\cos\theta}^{2}}-\frac{f_{f}L_{s}\dot{\theta}\cdot\left| \sin\theta\right|}{\left| \dot{\theta} \right|{\cos\theta}^{2}}\#\left( s15 \right) \end{aligned}$$

Therefore, the complete kinetic equation of the BM-TENG is:

$$\begin{aligned} \frac{d}{dt}\left( \frac{\delta L}{\delta\dot{\theta}} \right)-\frac{\delta L}{\delta\theta}=\frac{F_{e}L_{s}}{{\cos\theta}^{2}}-\frac{f_{f}L_{s}\dot{\theta}\cdot\left| \sin\theta\right|}{\left| \dot{\theta} \right|{\cos\theta}^{2}}\#\left( s16 \right) \end{aligned}$$

where $L=E_{k1}+E_{k2}+E_{k3}-E_{p1}-E_{p2}$, and $\frac{\dot{\theta}}{\left| \dot{\theta} \right|}$ means taking only the sign of $\dot{\theta}$.

**S1.2 Electrical modeling**

V-Q-x equation can be established to described the relationship between voltage, charge and separation distance of TENG. The equation is as follow:

$$\begin{aligned} V=-\frac{1}{C\left( x \right)}\times Q+V_{oc}\left( x \right)\#\left( s17 \right) \end{aligned}$$

Since the thickness of the dielectric film is much smaller than its longitudinal length, edge effects can be neglected when analyzing the capacitance between the electrodes. Edge effects refer to the increase in non-longitudinal components of the electric field formed inside the dielectric film by positive charges in the aluminum plates when the thickness of the dielectric film is large, resulting in a lower predicted model accuracy. Additionally, due to the thin thickness of the dielectric film, a parallel plate model can be used to describe the capacitance:

$$\begin{aligned} C=\frac{\varepsilon_{0}\varepsilon_{r}w\left( l-x \right)}{d}\#\left( s18 \right) \end{aligned}$$

where$\varepsilon_{0}$ is the vacuum permittivity, $\varepsilon_{r}$ is the relative permittivity of the dielectric film, $w$ is the width of the dielectric film, and $d$ is the thickness of the dielectric film.

For the idealized model, the charge distribution in the open-circuit can be predicted, the electric field inside the dielectric film can be calculated as:

$$\begin{aligned} E=\frac{\sigma x}{\varepsilon_{0}\varepsilon_{r}\left( l-x \right)}\#\left( s19 \right) \end{aligned}$$

Therefore, the open-circuit voltage is:

$$\begin{aligned} V_{oc}=Ed=\frac{\sigma xd}{\varepsilon_{0}\varepsilon_{r}\left( l-x \right)}\#\left( s20 \right) \end{aligned}$$

Thereby, the V-Q-x equation can be written as:

$$\begin{aligned} V=-\frac{d}{\varepsilon_{0}\varepsilon_{r}w\left( l-x \right)}\times Q+\frac{\sigma xd}{\varepsilon_{0}\varepsilon_{r}\left( l-x \right)}\#\left( s21 \right) \end{aligned}$$

According to Ohm's law $V=R\frac{dQ}{dt}$, solving the above equation yields:

$$\begin{aligned} R\frac{dQ}{dI}=-\frac{d}{\varepsilon_{0}\varepsilon_{r}w\left( l-x \right)}\times Q+\frac{\sigma xd}{\varepsilon_{0}\varepsilon_{r}\left( l-x \right)}\#\left( s22 \right) \end{aligned}$$

Solving the above formula can be obtained as:

$$\begin{aligned} I=\sigma wv\frac{d}{Rw\varepsilon_{0}\varepsilon_{r}v-d}\left\{ \frac{l}{l-vt}exp\left[ \frac{d}{Rw\varepsilon_{0}\varepsilon_{r}v}\ln\left( \frac{l-vt}{l} \right)-1 \right] \right\}\#\left( s23 \right) \end{aligned}$$

Thus, the output voltage of the in-plane sliding model can be expressed as:

$$\begin{aligned} V=\sigma wvR\frac{d}{Rw\varepsilon_{0}\varepsilon_{r}v-d}\left\{ \frac{l}{l-vt}exp\left[ \frac{d}{Rw\varepsilon_{0}\varepsilon_{r}v}\ln\left( \frac{l-vt}{l} \right)-1 \right] \right\}\#\left( s24 \right) \end{aligned}$$

**Section S2: Detailed structure of BM-TENG components.**


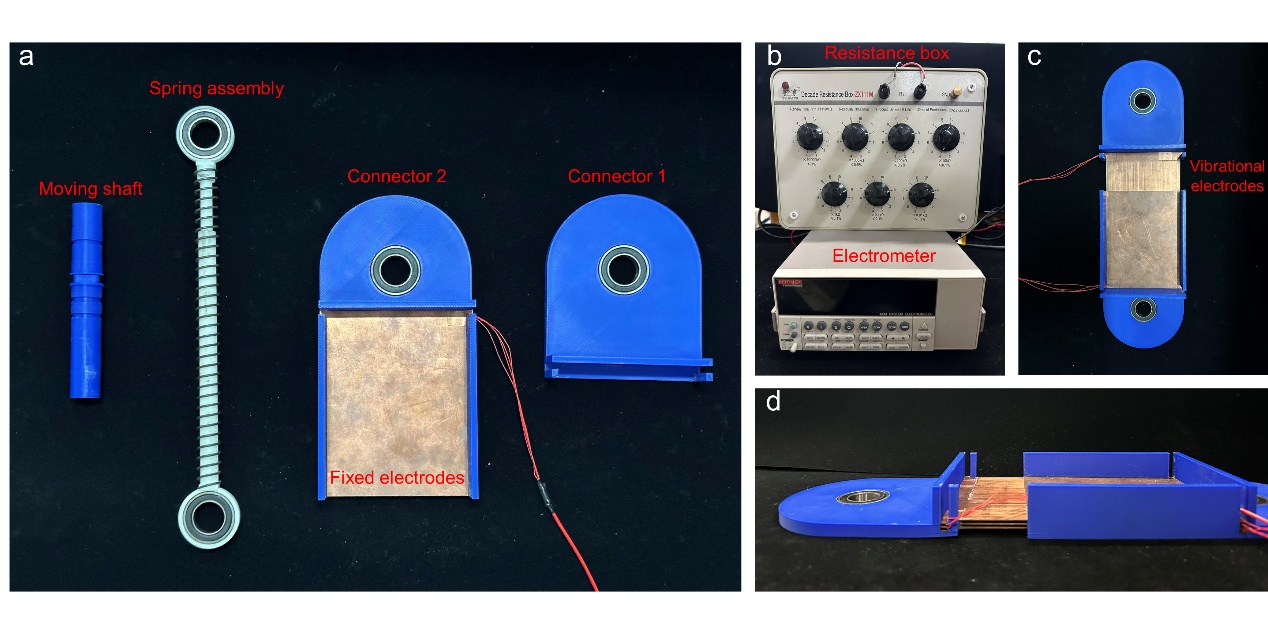


Figure S2. Detailed demonstration of BM-TENG components and experimental instruments.

In Figure S2. a, the moving shaft has three grooves, and these grooves hold the circlip. The groove in the middle of moving shaft is the guide groove for the support plate limit. Connectors 1-4 are connected with the moving shaft. The spring is set outside the connector 3,4 and is limited by the hook at the end of the connector 3,4. Connectors 1,2 are provided with grooves on both sides to place the electrode plates so that the electrode plates move with them. Figure S2. b exhibits detailed photo of electrometer and resistance box. In Figure S2. c and d, the front view and side view of the contact between the oscillating and fixed electrodes are shown. The multilayer structure can be clearly observed.

**Section S3: Experimental section**

**S3.1 Fabrication of the BM-TENG**

The BM-TENG consists of three primary components: the support base, the energy harvesting structure and the spring structure. The support base consists of a machined stainless steel plate (thickness: 4 mm; length of the groove: 570 mm), two stainless steel shafts (diameter: 20 mm) and four hydraulic buffer springs (maximum compressed distance: 40 mm). The energy harvesting structure consists of connector 1, connector 2, oscillating and fixed electrodes. The fixed electrode includes machined copper plates (thickness: 1 mm; length: 126 mm; width: 96 mm) and PTFE film (thickness: 130 $\mu$m). Two single-sided PTFE tape are pasted on both sides of the copper plate to form a single fixed electrode. The oscillating electrode only consists of machined copper plates. The spring structure is composed of commercial springs (outer diameter: 16 mm; inner diameter: 12.4 mm; 220 mm length in bistable system and 190 mm length in monostable system), connector 3 (outer diameter: 8 mm) and connector 4 (outer diameter: 11.8 mm; inner diameter: 9.2 mm). All the connectors and the moving shaft are made by 3D printing.

**S3.2 Experimental device**

In the experiment, linear motor (PBA X-Actuator-S800) is mainly used to simulate external sine and random excitation. An electrometer (6514, Keithley, USA) coupled with a data acquisition card (YGJ-DAQ-001) are used to precisely measure the voltage, current, charge of the system. The YGJ-6514 software can visualize and digitally filter the data. Resistance box (ZX111M) is used to test the optimal output power of the BM-TENG.
